# Anterior cervical decompression and fusion surgery for cervicogenic headache: A multicenter prospective cohort study

**DOI:** 10.3389/fneur.2022.1064976

**Published:** 2022-11-24

**Authors:** Liang Yang, Yongchao Li, Chen Dai, Xiaodong Pang, Duanming Li, Ye Wu, Xiongsheng Chen, Baogan Peng

**Affiliations:** ^1^Department of Orthopeadics, Featured Medical Center of Chinese People's Armed Police Forces, Tianjing, China; ^2^Department of Orthopeadics, The Third Medical Center of Chinese PLA General Hospital, Beijing, China; ^3^Department of Orthopeadics, Beijing 304th Hospital, Beijing, China; ^4^Department of Orthopeadics, Spine Center, Shanghai Changzheng Hospital, Second Affiliated Hospital of Naval Medical University, Shanghai, China

**Keywords:** anterior cervical decompression and fusion, cervical intervertebral disc degeneration, chronic neck pain, cervicogenic headache, cervical spondylosis

## Abstract

**Background:**

Cervicogenic headache (CEH) has long been recognized as a referred pain deriving from pathological changes in the upper cervical nerves. However, previous clinical studies found that anterior lower cervical discectomy for the treatment of cervical myelopathy and/or radiculopathy can also help relieve associated headaches. To date, there is still a lack of large sample and prospective study to investigate the effect of anterior cervical decompression and fusion (ACDF) on CEH associated with cervical spondylosis.

**Methods:**

A total of 656 patients with cervical radiculopathy and/or myelopathy were enrolled in three spinal centers. Among them, 221 patients who were diagnosed with CEH were collected in this study, and 204 completed a 1-year follow-up. The primary endpoint was headache intensity during a 12-month follow-up period measured by the numeric pain rating scale (NPRS). The secondary outcome measures included headache frequency, headache duration, and the neck disability index (NDI).

**Results:**

Among all 204 patients with CEH who completed a 1-year follow-up, 166 received anterior cervical surgery (surgery group) and 38 received conservative treatment (conservative group). There were statistically significant lower NPRS in the surgical group during follow-up. Between-group differences showed that NPRS in the surgery group was significantly greater improvement at 1 month (2.8, 95% CI: 2.0, 3.6), 3 months (2.6, 95% CI: 1.8, 3.4), 6 months (2.4, 95% CI: 1.6, 3.2), and 12 months (1.5, 95% CI: 0.7, 2.4) (*p* < 0.05 for all). There were statistically significant lower NDI, less frequent headaches, and lower headache duration in the surgery group during follow-up (*p* < 0.05 for all).

**Conclusion:**

This study indicates that ACDF can effectively relieve CEH associated with cervical myelopathy and/or radiculopathy.

## Introduction

Cervicogenic headache (CEH) is a common chronic and recurrent headache (1). It is thought to be painful in the head, but in fact, the lesion is in the neck. In the general population, the prevalence of CHE varies with different diagnostic criteria. It is estimated that 1–4.1% of the population may experience CEH (2). According to the definition of the International Headache Society (IHS) for CEH in 2013 (3) and 2018 (4), CEH is defined as any headache caused by the disturbance of the cervical spine or its components, such as disc, bone, and/or soft tissue elements, usually but not necessarily accompanied by neck pain.

The specific pathogenesis of CEH is poorly understood. It has long been considered a referred pain caused by pathological changes in the upper cervical nerves (C1–C3) (2). However, as we all know, cervical spondylosis occurs mostly in the lower cervical spine (C4–C7) and rarely in the upper cervical spine (5, 6). In reality, Diener et al. (7) and later Persson et al. (8) identified the role of the lower cervical spine as a potential source of CEHs in the early 21st century. Meanwhile, a large number of clinical studies (5, 7, 9–15), including *post-hoc* analysis from a multicenter randomized clinical trial (14), found that anterior lower cervical discectomy for the treatment of cervical myelopathy and/or radiculopathy also helps to relieve associated headaches, and the effect can be maintained up to a 7-year (14) and 10-year follow-up (13). However, the diagnosis of CEH in the above clinical studies is not based on any pre-existing criteria. Headache related to cervical spondylosis is not equal to “cervicogenic headache” (16). It is uncertain how many patients with cervical spondylosis have headaches that meet the most up-to-date diagnostic criteria for CEH—the International Classification of Headache Disorders, third edition (ICHD-3) (3, 4). According to the previous literature, the prevalence of headaches associated with cervical spondylosis ranges from 86 to 88%, and the prevalence of severe headache is about 52% (5, 9). However, according to a retrospective study (6) and a small sample prospective study (17), the incidence rate of cervical spondylosis patients with CEH (based on ICHD-3 beta version) was only 21.4% (15/70) and 30% (50/166). Therefore, it is difficult to exclude the interference of headache from other causes and determine whether anterior cervical surgery is helpful to improve CEH associated with cervical spondylosis.

Recently, a retrospective study found that anterior lower cervical discectomy can also relieve the accompanying CEH based on the criteria of the ICHD-3 beta version (6). So far, there is a lack of large sample and prospective study to investigate the effect of anterior cervical decompression and fusion (ACDF) surgery on CEH associated with cervical spondylosis. To this end, we conducted a multicenter prospective study to determine whether ACDF for cervical radiculopathy and/or myelopathy can also help relieve related CEH based on the criteria of the ICHD-3 beta version.

## Materials and methods

### Ethics approval

The study was approved by the Ethics Committee of the Third Medical Center of Chinese PLA General Hospital and written informed consent was obtained from each participant.

### Study population

We conducted a multicenter, prospective, cohort clinical trial in three centers from August 2017 to August 2018. This trial was prospectively registered in the Chinese Clinical Trials Registry (ChiCTR-ONC-17012027). Originally, the purpose of our clinical trial was to observe the effect of ACDF on tinnitus in patients with cervical spondylosis. During this period, we simultaneously observed the effect of ACDF on CHE in the same group of patients. A total of 656 patients with cervical radiculopathy and/or myelopathy were enrolled in three spinal centers. They did not respond to conservative treatment for at least 3 months and were candidates for ACDF surgery due to severe neurological dysfunction or intolerable symptoms. Among them, 221 patients who were diagnosed with CEH according to the ICHD-3 beta version criteria (Table 1) (3) were collected in this study. A total of 178 (80.5%) patients underwent ACDF (surgery group). The remaining 43 patients refused surgery due to fear or other reasons and continued to receive conservative treatment (conservative group). In all, 204 (92.3%) patients were followed up for 1 year, including 166 (93.3%) cases in the surgery group and 38 (88.4%) cases in the conservative group.

**Table 1:** The diagnostic criterions recommended by the international classification of headache disorders, ICHD 3rd edition.

A. Any headache fulfilling criterion C
B. Clinical, laboratory and/or imaging evidence of a disorder or lesion within the cervical spine or soft tissues of the neck, known to be able to cause headache
C. Evidence of causation demonstrated by at least two of the following: 1. headache has developed in temporal relation to the onset of the cervical disorder or appearance of the lesion 2. headache has significantly improved or resolved in parallel with improvement in or resolution of the cervical disorder or lesion 3. cervical range of motion is reduced and headache is made significantly worse by provocative maneuvers 4. Headache is abolished following diagnostic blockade of a cervical structure or its nerve supply
D. Not better accounted for by another ICHD-3 diagnosis

The inclusion criteria were patients between the ages of 18 and 55 years with typical signs and symptoms of myelopathy and/or radiculopathy; those with objective signs of the spinal cord and/or nerve root compression as shown on magnetic resonance imaging (MRI); and those having concomitant CEH with or without other symptoms, such as dizziness, tinnitus, blurred vision, and nausea.

Exclusion criteria included patients with a history of neck trauma or surgery; those suffering from neurological disease or any other possible treatable causes for headache; and those with congenital or developmental cervical malformations and those unable to follow the study.

### Treatment

Decompression and fusion segments depended on clinical manifestations and corresponding nerve root and/or spinal cord compressions shown on MRI. The choice of surgery levels was determined by a senior spine surgeon at each center (BP, YW, and XC, respectively). Anterior cervical interbody fusion was performed with a cage that was filled up with autogenous bone obtained by local decompression and anterior plate fixation. The operative segments ranged from C2/3 to C7/T1 (Table 2). Conservative treatment included intermittent fixation of the cervical collar, physiotherapy, and oral medications including non-steroidal anti-inflammatory drugs, muscle relaxants, and analgesics.

**Table 2:** Comparison of participant characteristics of the 2 treatment groups at baseline.

**Characteristic**	**Surgery group (*n* = 166)**	**Conservative group (*n* = 38)**	***P***
Sex, female, *n* (%)	95 (57.2%)	21 (55.3%)	0.83
Age (y)	45.2 (7.9)	46.8 (5.5)	0.21
Headache intensity (NPRS)	6.2 (1.7)	5.9 (1.6)	0.28
Disability (NDI)	20.9(8.2)	19.1(8.0)	0.83
Headache frequency	4	4	0.18
Headache duration	3	3	0.18
CSM	89 (53.6%)	21 (55.3%)	
CSR	33 (19.9%)	6 (15.8%)	
Mixed CS	44 (26.5%)	11 (28.9%)	
**Diseased level**	*n* = 272	*n* = 67	
C2/3	3 (1.1%)	1 (1.5%)	
C3/4	20 (7.4%)	4 (6.0%)	
C4/5	59 (21.7%)	16 (23.9%)	
C5/6	105 (38.6%)	28 (41.8%)	
C6/7	80 (29.4%)	17 (25.3%)	
C7/T1	5 (1.8%)	1 (1.5%)	

Data are mean (SD), median, or number (%). NPRS, numeric pain rating scale; NDI, Neck Disability Index; CS, cervical spondylosis; CSM, cervical spondylotic myelopathy; CSR, cervical spondylotic radiculopathy; Mixed CS, Mixed cervical spondylosis. Comparison of means among two groups (significant at *P* < 0.05).

### Outcome measures

The primary efficacy endpoint was the mean change in headache intensity from baseline to 12 months in the surgery group, as measured by the Numeric Pain Rating Scale (NPRS), compared with the mean change in the conservative group. At baseline, 1, 3, 6, and 12 months after treatment, patients were asked to use an 11-point scale ranging from 0 (“no pain”) to 10 (“worst pain imaginable”) to express the average headache intensity in the past week (18). The NPRS is a reliable and effective tool for assessing pain intensity (19).

The study was also powered for the assessment of secondary efficacy endpoints: the neck disability index (NDI), headache frequency, and headache duration. The NDI is the most widely used tool to assess self-rated disability in patients with neck pain (20), a self-report questionnaire with 10 items scored from 0 (no disability) to 5 (complete disability) (21). The numeric responses for each item are summed, giving a total score between 0 and 50. Headache frequency was measured as the number of days with headache in the last week, ranging from 0 to 7 days. Headache duration was the total hours of headache in the last week and had six possible ranges, i.e., (1) 0–5 h, (2) 6–10 h, (3) 11–15 h, (4) 16–20 h, (5) 21–25 h, or (6) 26 h or more.

Baseline data included age, gender, NPRS score, NDI score, headache frequency, headache duration, diseased disc segments, and classification of cervical spondylosis were collected before treatment. Re-evaluation was performed at 1, 3, 6, and 12 months, respectively, after treatment.

### Statistical analysis

Descriptive statistics, including frequency counts for categorical variables and measures of central tendency and dispersion for continuous variables, were calculated to summarize the data. Comparisons of NPRS score and NDI score between the two groups were at baseline, 1, 3, 6, and 12 months conducted with a one-way analysis of variance. Comparisons of NPRS score and NDI score at baseline, 1, 3, 6, and 12 months within groups were conducted with paired-sample *t*-test. Separate Mann–Whitney *U* tests were performed with the headache frequency and headache duration between the two groups at baseline, 1, 3, 6, and 12 months. SPSS version 23.0 (IBM, Quarry Bay, Hong Kong) was used for all analyses. The significance level was set at *p* < 0.05.

## Results

After cervical decompression, NPRS of the surgery group decreased immediately (6.2 ± 1.7 at baseline, 2.1 ± 1.5 at 1 month, *p* < 0.001) and lasted for 12 months (2.4 ± 1.6 at 12 months, *p* < 0.001). After conservative treatments, NPRS of the conservative group also decreased (5.9 ± 1.6 at baseline, 4.7 ± 1.5 at 1 month, *p* = 0.002) and lasted for 12 months (3.6 ± 1.7 at 12 months, *p* < 0.001). At 1, 3, 6, and 12 months after treatment, NPRS of the surgery group was significantly lower than that of the conservative group (Table 3, *p* < 0.05). The improvement of NPRS in the surgery group was significantly greater than that in the conservative group. The difference in NPRS improvement between the two groups was 2.8 at 1 month, 2.6 at 3 months, 2.4 at 6 months, and 1.5 at 12 months (Table 3, *p* < 0.05). Besides, in order to explore the influence of surgical segments on headache improvement, we classified the surgery patients into two subgroups, namely, single segment (*n* = 80, 48.2%) and multisegment (more than 2 segments, *n* = 86, 51.8%) and compared the NPRS between the two subgroups at baseline and 12 months. There was no significant difference in NPRS between the two subgroups (6.2 ± 1.7 and 6.2 ± 1.7 at baseline, *p* = 0.849; 2.3 ± 1.6 and 2.5 ± 1.6 at 12 months, *p* = 0.644).

**Table 3:** Changes in headache intensity (NPRS) and disability (NDI) with 95% confidence intervals for both groups and between-group differences.

**Variable**	**Surgery**	**Conservative**	**Between-group differences**
**Headache intensity (NPRS 0–10)**			
Baseline: mean (SD)	6.2 (1.7)	5.9 (1.6)	
1-Month: mean (SD)	2.1 (1.5)	4.7 (1.5)	
Change Score: baseline to 1-Month	4.0 (3.7, 4.4)	1.2 (0.5, 1.9)	2.8 (2.0, 3.6); *P* < 0.001
3-Month: mean (SD)	2.1 (1.3)	4.4 (1.2)	
Change Score: baseline to 3-Month	4.1 (3.7, 4.4)	1.5 (0.8, 2.2)	2.6 (1.8, 3.4); *P* < 0.001
6-Month: Mean (SD)	2.1 (1.3)	4.2 (1.1)	
Change Score: Baseline to 6-Month	4.1 (3.7, 4.4)	1.7 (1.0, 2.3)	2.4 (1.6, 3.2); *P* < 0.001
12-Month: mean (SD)	2.4 (1.6)	3.6 (1.7)	
Change Score: baseline to 12-Month	3.8 (3.4, 4.1)	2.3 (1.4, 3.1)	1.5 (0.7, 2.4); *P* < 0.001
Disability (NDI 0–50)			
Baseline: mean (SD)	20.9 (8.2)	19.1 (8.0)	
1-Month: mean (SD)	8.8 (4.6)	13.0 (7.4)	
Change score: baseline to 1- Month	12.1 (10.6, 13.6)	6.1 (2.2, 10.0)	6.0 (2.4, 9.6); *P* = 0.001
3-Month: mean (SD)	8.3 (4.0)	13.0 (6.7)	
Change score: baseline to 3- Month	12.7 (11.2, 14.1)	6.2 (2.5, 9.8)	6.5 (3.0, 10.0); *P* < 0.001
6-Month: mean (SD)	7.8 (3.4)	13.3 (6.1)	
Change score: baseline to 6- Month	13.1 (11.8, 14.5)	5.8 (2.4, 9.2)	7.3 (4.1, 10.6); *P* < 0.001
12-Month: mean (SD)	7.2 (4.5)	13.5 (7.1)	
Change score: baseline to 12-Month	13.7 (12.3, 15.1)	5.6 (2.2, 9.0)	8.1 (4.7, 11.5); *P* < 0.001

NPRS, Numeric Pain Rating Scale, 0–10, lower scores indicate less pain; NDI, Neck Disability Index, 0–50, lower scores indicate greater function.

During the follow-up, the NDI in the surgical group was significantly lower than that in the conservative group (Table 3, *p* < 0.05). The NDI in the surgical group was 20.9 and 7.2 at baseline and 12 months, respectively, while that in the conservative group was 19.1 and 13.5 at baseline and 12 months, respectively. Between-group differences showed a significantly greater improvement in the NDI at 1 month (6.0, 95% CI: 2.4, 9.6), 3 months (6.5, 95% CI: 3.0, 10.0), 6 months (7.3, 95% CI: 4.1, 10.6), and 12 months (8.1, 95% CI: 4.7, 11.5) in the surgery group (Table 2, *p* < 0.05).

The Mann–Whitney *U* test showed that the incidence of headache was lower in the surgery group than in the conservative group at 1, 3, 6, and 12 months (*p* < 0.001). The duration of headache in the surgery group was significantly lower at 1, 3, 6, and 12 months (Table 4, *p* < 0.001).

**Table 4:** All outcome measures over 12 months for each treatment group.

**Measure**	**Group**	**Baseline**	**1 Month**	**3 Months**	**6 Months**	**12 Months**
Headache intensity (NPRS 0–10): mean (SD)	SG	6.2 (1.7)	2.1 (1.5)	2.1 (1.3)	2.1 (1.3)	2.4 (1.6)
	CG	5.9 (1.6)	4.7 (1.5)	4.4 (1.2)	4.2 (1.1)	3.6 (1.7)
Disability (NDI 0–50): mean (SD)	SG	20.9 (8.2)	8.8 (4.6)	8.3 (4.0)	7.8 (3.4)	7.2 (4.5)
	CG	19.1 (8.0)	13.0 (7.4)	13.0 (6.7)	13.3 (6.1)	13.5 (7.1)
Headache frequency (0–7 days): median	SG	4	2	2	2	2
	CG	4	3	3	3	3
Headache duration: median	SG	3	1	1	1	1
	CG	3	2	2	2	2

A total of 11 patients had mild dysphagia and nine patients had hoarseness immediately after the operation, but all of the symptoms disappeared within 1 month. Notably, five patients developed C5 nerve root paralysis, and the symptoms disappeared within 3 months after the operation. There were no other surgical complications, such as aggravation of neurological symptoms, implant loosening and loss, and infection.

## Discussion

Numerous studies have identified the short-, mid-, and long-term effects of anterior cervical surgery on headaches associated with cervical myelopathy and/or radiculopathy (9, 11, 13, 14); however, headache related to cervical spondylosis is not equal to “cervicogenic headache” (16). As primary headaches are often associated with neck pain, it is difficult to distinguish primary headache from real CEH only according to a single headache score rather than based on systematic diagnostic criteria (16). Rinna et al. (9) reported that the prevalence of headache in a large sample of patients with cervical spondylosis is 86% (865/1,003). Similarly, Schrot et al. (5) reported that the prevalence of headache is 88% (228/260). The high prevalence of headaches in these two reports may be related to the fact that headaches have not been diagnosed according to any ICHD criteria of CEH. According to the ICHD-3 beta criteria, Shimohata et al. (17) reported that the prevalence of CEH with cervical spondylosis is 21.4% (15/70). The low prevalence of headaches may be attributable to the small sample size. In this study, we found that the prevalence of CEH in the patients with cervical radiculopathy and/or myelopathy is 33.7% (221/656). Since we strictly followed the ICHD-3 beta criteria for CEH diagnosis and this was a large-sample, multicenter, prospective study, this prevalence can be more reasonable and credible.

This study has also shown that CEH in patients with cervical radiculopathy and/or myelopathy significantly improved or disappeared after ACDF, which was consistent with previous studies (6, 17, 22). To the best of our knowledge, to date, no large-scale, prospective, multicenter study has examined the efficacy of ACDF to relieve CEH diagnosed based on the ICHD-3 beta version. Although the headache of the conservative treatment group improved after treatment, the curative effect of the surgery group was significantly better than that of the conservative treatment group. Between-group differences showed a statistically significantly greater improvement in the NPRS at 1, 3, 6, and 12 months in the surgery group. There were statistically significant lower NDI, less frequent headaches, and lower headache duration in the surgery group during follow-up. As the patients we selected have severe neurological dysfunction or intolerable symptoms, it is obvious that a randomized controlled trial is not feasible in logic and ethics. However, we compared the effect of patients who did not agree with the surgery (conservative group) with the surgery group, so this conclusion can be more reliable.

**Figure 1:**
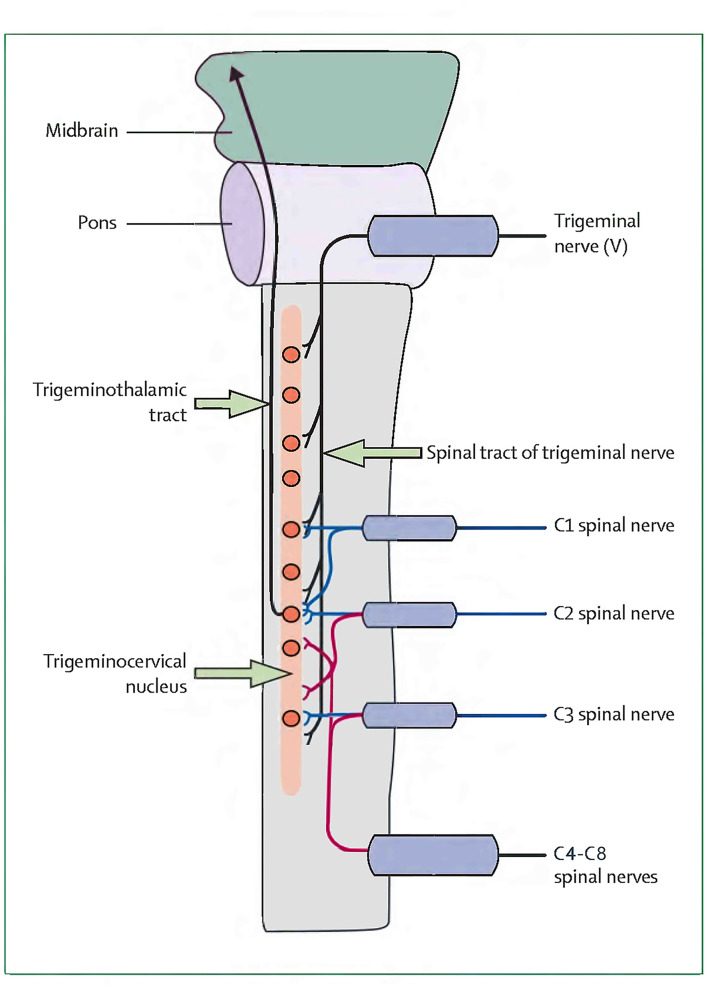
A sketch of the headache referral from C1 to C8 spinal nerves.

Currently, less is known about the pathogenesis of CEHs, and their anatomical pain generators are even more difficult to define (13). Bogduk and Govind (2) found that CEH is a referred pain originating from lesions of the upper cervical nerves. Nociceptive afferents of the C1–C3 nerves and the trigeminal nerve converge on the trigeminocervical nucleus in the superior cervical spinal cord. This convergence mediates the transmission of pain signals from the neck to areas of the head innervated by the cervical or trigeminal nerve (2). However, this theory is difficult to explain the mechanism of headache caused by lower cervical spine pathology (5). The concept of CEH originating from the lower cervical spine was first proposed by Diener et al. (7), who found that CEHs in 80% of patients with lower cervical disc herniation (below C4) improved or disappeared after surgery. Later, Persson et al. (8) analyzed headaches in patients with lower cervical radiculopathy. Selective nerve root block at the pathological level resulted in headache reduction of 50% or more, with 69% of patients reporting complete headache relief. Therefore, Diener and Persson inferred that nociceptive afferent from the lower cervical roots also converged on the trigeminocervical nucleus (7, 8). The nerve distribution of the cervical intervertebral disc is similar to that of the lumbar disc. It is innervated multisegmentally. Fujimoto et al. (23) reported that the C5–C6 disc in rats is innervated multisegmentally by C2–C8 dorsal root ganglia (DRG) neurons, which indicates that the trigeminocervical nucleus may receive not only C1–C3 spinal nerve afferents but also partly C4–C8 nerve afferents by the ways of C2 and C3 DRG. This study found that cervical spondylosis of the lower cervical spine can also cause CEH, and ACDF surgery can significantly improve CEH, suggesting that nociceptive afferents from lower cervical nerves may converge to trigeminocervical nucleus. Bogduk et al. (2) originally depicted a very vivid sketch of the convergence of the upper cervical spine nerves (C1–C3) and the trigeminal nerve in the trigeminocervical nucleus. On the basis of Bogduk et al., we redrew the convergence diagram of the whole cervical nerves (C1–C8) and the trigeminal nerve in the trigeminocervical nucleus in Figure 1. Further anatomical and neurophysiological studies are needed to better define and verify the relationship between the inferior cervical nerve and the trigeminocervical nucleus, as well as the pathogenesis of CEH.

The most typical pathological change in cervical spondylosis is intervertebral disc degeneration (24). Degenerative cervical intervertebral disc has long been recognized as a common source of chronic neck pain (25–29). Recent studies by Yang et al. (30) and Wu et al. (31) found significantly increased numbers of substance P-positive nerve fibers deeply ingrown into the degenerative cervical discs in patients with severe neck pain compared with discs from cervical radiculopathy or myelopathy patients without neck pain or mild neck pain and normal control discs. The convincing evidence of neck pain caused by cervical disc degeneration is that neck pain was significantly reduced or disappeared immediately after the injection of local anesthetics into the degenerative disc (30–32). Therefore, we believe that inflammation caused by cervical disc degeneration may stimulate nociceptors in the cervical intervertebral disc, resulting in neck pain. At the same time, these nociceptive excitabilities are projected into the trigeminocervical nucleus in the upper cervical spinal cord, resulting in CEH. In fact, any irritation or compression of cervical nerve root or spinal cord caused by cervical radiculopathy or myelopathy may also affect the nociceptive afferents of the diseased cervical intervertebral disc and then aggravate headache (10, 22). Therefore, we believe that ACDF may improve CEH by removing the degenerative cervical disc and its internal nociceptors and decompressing the cervical nerve root or spinal cord. In addition, posterior laminoplasty can also relieve CEH by indirectly decompressing the spinal cord (10, 17). However, laminoplasty is less durable than anterior cervical fusion for headache relief (17).

There are other hypotheses in the literature to explain the etiology of CEH in the lower cervical spine. Bogduk and Govind (2) suggested that there is no direct connection between the lower cervical afferents and the trigeminocervical nucleus, but intermediate mechanisms may be involved, such as secondary spinal kinesthesia and muscle tension affecting the upper cervical joints. Headache relief may differ between anterior arthroplasty and fusion if spinal kinesthesia is the specific mechanism (5). A *post-hoc* analysis of a prospective, multicenter study with a 10-year follow-up conducted by Lombardi et al. (13) found that both arthroplasty and ACDF were effective in relieving headaches associated with cervical radiculopathy and/or myelopathy, but the arthroplasty group had lower headache scores than the ACDF group. This result supports a role for the preservation of spine kinematics in the pathogenesis of CEH. Thind et al. (14) also found the same results as Lombardi et al. in a 7-year follow-up study. However, a similar study by Schrot et al. (5) compared ACDF and arthroplasty in single-level cervical spondylosis with 2-year follow-up data. Similarly, they found significant relief from CEH, but unlike previous studies, there was no significant difference between the surgery groups. Sun et al. (10) also found the same results as Schrot et al. in a retrospective study. Thus, postoperative spinal kinesthetic improvement may be less important in headache relief (5). The key to the above-mentioned controversial results is that the studies have different diagnostic criteria, and none of them are used to diagnose CEH in accordance with the ICHD-3 criteria. At the same time, interference from other causes of headache cannot be ruled out.

Other proposed mechanisms are sinuvertebral nerve (SVN) or sympathetic nerve irritation at the uncovasculoradicular junction, anterior dura mater, or cervical posterior longitudinal ligament (PLL). The cervical dura and PLL have different sympathetic innervation and may induce sympathetic reflexes (33). The activity may pass through the ganglia and the sympathetic trunk to the trigeminocervical nucleus, subsequently inducing CEH (22). In addition, Thind et al. (14) proposed that SVN irritation at the uncovasculoradicular junction and anterior dura may be the cause of CEH. Since the inferior branch of the SVN can reach three segments below its origin, nociception from the lower cervical segment, such as C6, can project to the third cervical nerve and ultimately to the trigeminocervical nucleus, leading to CEH. Indeed, both anterior cervical surgery and posterior decompression can relieve headache (5, 10, 17). Sun et al. (10) found that ACDF, arthroplasty, and laminoplasty can all significantly alleviate headache and believed that the headache associated with cervical spondylosis may be the result of the compression of the spinal cord itself. In addition, innervation of the dorsal dura is relatively sparse compared to the ventral dura (5). Therefore, headache development and relief are more likely to be associated with spinal cord injury rather than stimulation of the SVN or sympathetic nerves on the ventral dura, uncovasculoradicular junction, or PLL.

In addition, Schrot et al. (5) found that headache relief was not related to the level of surgery. These findings are consistent with our research. In the surgical group, only 23 (8.5%) surgical segments were the upper cervical spine (C2–C3 or C3–C4). When the above cases were eliminated, the remaining cases in the surgical group still achieved consistent results.

This study has several limitations. First, in this study, the patients were selected for their radiculopathy and/or myelopathy rather than headaches. Therefore, the results cannot be interpreted as all patients with cervical spondylosis complicated by CEH should be treated with anterior cervical decompression surgery. Second, the pathogenesis of CEH may be multifactorial. Most of the studies related to CEH, including this study, are clinical studies and cannot provide direct and convincing evidence to confirm the pathogenesis of CEH. However, this study provides a direction for relevant basic research. Finally, this study only completed a 1-year follow-up, and further long-term follow-up is needed to confirm the efficacy of ACDF in the treatment of CEH.

## Conclusion

This study indicates that both ACDF and conservative treatment can improve CEH associated with cervical myelopathy and/or radiculopathy, but ACDF is better than conservative treatment.

## Data availability statement

The raw data supporting the conclusions of this article will be made available by the authors, without undue reservation.

## Ethics statement

The studies involving human participants were reviewed and approved by the Local Ethics Committee of the Third Medical Center of Chinese PLA General Hospital. The patients/participants provided their written informed consent to participate in this study. Written informed consent was obtained from the individual(s) for the publication of any potentially identifiable images or data included in this article.

## Author contributions

BP and XC designed the study. LY, YL, and BP drafted the manuscript. LY, XP, YW, and XC carried out data analysis. LY, CD, XP, DL, YW, and XC performed data collection. YL, XP, YW, XC, and BP revised the manuscript. All authors read and approved the final manuscript. All authors contributed to the article and approved the submitted version.

## Conflict of interest

The authors declare that the research was conducted in the absence of any commercial or financial relationships that could be construed as a potential conflict of interest.

## Publisher's note

All claims expressed in this article are solely those of the authors and do not necessarily represent those of their affiliated organizations, or those of the publisher, the editors and the reviewers. Any product that may be evaluated in this article, or claim that may be made by its manufacturer, is not guaranteed or endorsed by the publisher.
